# Long-term cardiotoxicity in germ cell cancer survivors after platinum-based chemotherapy: cardiac MR shows impaired systolic function and tissue alterations

**DOI:** 10.1007/s00330-023-10420-w

**Published:** 2023-11-20

**Authors:** Antonia Beitzen-Heineke, Christina Charlotte Rolling, Christoph Seidel, Jennifer Erley, Isabel Molwitz, Kai Muellerleile, Dennis Saering, Juliana Senftinger, Niklas Börschel, Nils Wolfgang Engel, Carsten Bokemeyer, Gerhard Adam, Enver Tahir, Hang Chen

**Affiliations:** 1 Department for Oncology, Hematology and Bone Marrow Transplantation with the Section of Pneumology, University Medical Center Hamburg Eppendorf, Martinistr. 52, 20246 Hamburg, Germany; 2 Department of Diagnostic and Interventional Radiology and Nuclear Medicine, University Hospital Hamburg Eppendorf, Hamburg, Germany; 3 Department of General and Interventional Cardiology, University Heart and Vascular Center Hamburg, Hamburg, Germany; 4 Information Technology and Image Processing, University of Applied Sciences Wedel, Wedel, Germany

**Keywords:** Platinum, Cardiotoxicity, Testicular neoplasms, Survivorship, Multiparametric magnetic resonance imaging

## Abstract

**Objectives:**

Long-term toxicities of germ cell cancer (GCC) treatment are of particular importance in young men with a life expectancy of several decades after curative treatment. This study aimed to investigate the long-term effects of platinum-based chemotherapy on cardiac function and myocardial tissue in GCC survivors by cardiac magnetic resonance (CMR) imaging.

**Methods:**

Asymptomatic GCC survivors ≥ 3 years after platinum-based chemotherapy and age-matched healthy controls underwent CMR assessment, including left ventricular (LV) and right ventricular (RV) ejection fraction (EF), strain analysis, late gadolinium enhancement (LGE) imaging, and T1/T2 mapping.

**Results:**

Forty-four survivors (age 44 [interquartile range, IQR 37–52] years; follow-up time 10 [IQR 5–15] years after chemotherapy) and 21 controls were evaluated. LV- and RVEF were lower in GCC survivors compared to controls (LVEF 56 ± 5% *vs.* 59 ± 5%, *p* = 0.017; RVEF 50 ± 7% *vs.* 55 ± 7%, *p* = 0.008). Seven percent (3/44) of survivors showed reduced LVEF (< 50%), and 41% (18/44) showed borderline LVEF (50–54%). The strain analysis revealed significantly reduced deformation compared to controls (LV global longitudinal strain [GLS] -13 ± 2% *vs.* -15 ± 1%, *p* < 0.001; RV GLS -15 ± 4% *vs*. -19 ± 4%, *p* = 0.005). Tissue characterization revealed focal myocardial fibrosis in 9 survivors (20%) and lower myocardial native T1 times in survivors compared to controls (1202 ± 25 ms *vs.* 1226 ± 37 ms, *p* = 0.016). Attenuated LVEF was observed after two cycles of platinum-based chemotherapy (54 ± 5% *vs.* 62 ± 5%, *p* < 0.001).

**Conclusion:**

Based on CMR evaluation, combination chemotherapy with cumulative cisplatin ≥ 200 mg/m^2^ is associated with attenuated biventricular systolic function and myocardial tissue alterations in asymptomatic long-term GCC survivors.

**Clinical relevance statement:**

Platinum-based chemotherapy is associated with decreased systolic function, non-ischemic focal myocardial scar, and decreased T1 times in asymptomatic long-term germ cell cancer survivors. Clinicians should be particularly aware of the risk of cardiac toxicity after platinum-based chemotherapy.

**Key Points:**

• *Platinum-based chemotherapy is associated with attenuation of biventricular systolic function, lower myocardial T1 relaxation times, and non-ischemic late gadolinium enhancement.*

• *Decreased systolic function and non-ischemic late gadolinium enhancement are associated with a cumulative cisplatin dose of * ≥ *200 mg/m*^*2*^*.*

• *Cardiac MRI can help to identify chemotherapy-associated changes in cardiac function and tissue in asymptomatic long-term germ cell cancer survivors.*

**Graphical Abstract:**

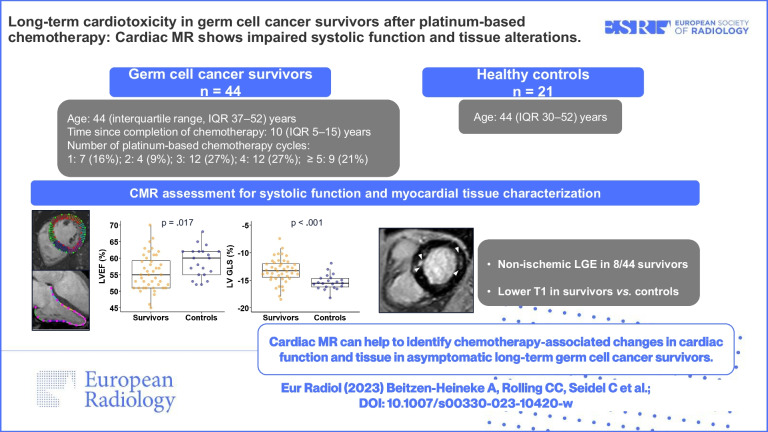

**Supplementary Information:**

The online version contains supplementary material available at 10.1007/s00330-023-10420-w.

## Introduction

Germ-cell cancer (GCC) is the most common cancer in young men aged between 20 and 40 years. Due to an excellent sensitivity to platinum-based chemotherapy and the use of multimodal treatment approaches using chemotherapy and secondary tumor resection, cure rates of more than 90% can be achieved even in metastatic disease [1–3]. As a result, long-term toxicities after GCC treatment are of particular importance in this group of cancer survivors with a life expectancy of several decades.

Long-term sequelae of chemo- and radiotherapy for GCC include neuro-, pulmonary, and renal toxicity, an increased risk of secondary malignancy, hypogonadism, and infertility. Furthermore, GCC survivors are at increased risk for metabolic syndrome and cardiovascular disease [4]. Risk estimates for cardiovascular disease range from 1.3- to 5.7-fold after platinum-based chemotherapy compared to surgery only [5–8]. Therefore, optimization of cardiovascular risk factors is recommended in GCC survivorship care. However, knowledge about cardiac function and myocardial alterations in GCC survivors is limited, and no specific recommendations for cardiological examinations in GCC survivors do exist. Previous studies investigating cardiac function of GCC survivors using echocardiography described impaired diastolic function but no impairment of systolic function, except for one study that described decreased left ventricular ejection fraction (LVEF) in one out of 37 survivors after platinum-based treatment [9–13].

Echocardiography is widely used to monitor cardiac function in patients undergoing potentially cardiotoxic cancer treatment. Using strain analysis, early changes in cardiac contractility can be detected before LVEF changes are manifest [14, 15]. Strain imaging assesses the myocardial deformation capacity by quantifying the shortening of a myocardial segment in percent from end-diastole to end-systole. The assessment of LVEF and global longitudinal strain (GLS) using echocardiography is recommended in patients receiving a potentially cardiotoxic treatment [16, 17]. LV global circumferential strain (GCS) is also predictive of chemotherapy-related cardiotoxicity but has not been established as a screening parameter [18]. In case of insufficient imaging quality, cardiac magnetic resonance (CMR) imaging, which represents the gold standard for quantification of cardiac function and volumes, is recommended as an alternative imaging modality in evaluating cardiotoxicity [16, 19, 20]. The characterization of myocardial tissue by CMR may provide further information on cardiac changes during oncological treatment [19]. T2 mapping identifies tissue inflammation characterized by edema, and T1 mapping is used to detect interstitial fibrosis. Late gadolinium enhancement (LGE) can identify myocardial fibrosis and further differentiate ischemic from non-ischemic scar tissue based on the LGE patterns [20].

Therefore, this study aimed to gain insights into the long-term cardiac effects of platinum-based chemotherapy by characterizing cardiac function and myocardial tissue in GCC survivors using CMR imaging.

## Methods

### Study population

**Fig. 1:**
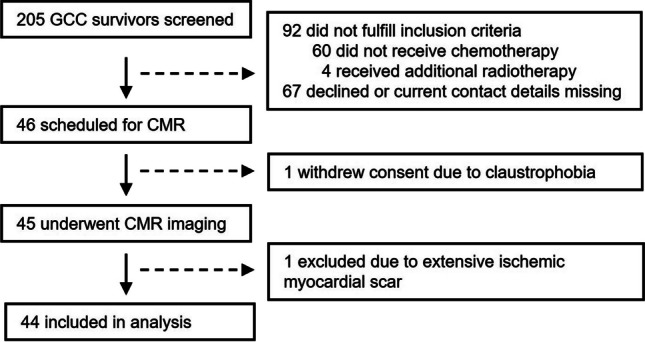
Flow chart of recruitment of study participants. Flow chart presenting inclusion and exclusion of 205 patients who presented for routine follow-up after treatment for germ cell cancer

The study was approved by the local ethics committee (PV7173) and carried out in accordance with the Declaration of Helsinki. All participants gave written informed consent. Forty-six asymptomatic consecutive men with a history of GCC ≥ 3 years after completion of platinum-based chemotherapy were prospectively recruited between March 2020 and January 2022 and underwent a CMR scan. Survivors who had received radiotherapy were excluded. The general exclusion criteria were MRI contraindications, any history of coronary artery disease, heart failure, and arrhythmia. A historical cohort of 21 age-matched healthy men was used as a control cohort. One participant from the survivor group was excluded from the analysis because of extensive ischemic myocardial fibrosis due to silent myocardial infarction, and one patient withdrew consent due to claustrophobia (Fig. 1).

Anamnestic cardiovascular risk factors and medication were documented, and a laboratory analysis was performed, including NT-proBNP, Troponin I, cholesterol, low-density lipoprotein (LDL), high-density lipoprotein (HDL), glomerular filtration rate (GFR), creatinine, and testosterone levels.

### CMR protocol

CMR was performed on a 3.0-T scanner (Ingenia, Philips Medical Systems) as previously described [21]. Imaging was performed using a standard retrospective ECG-triggered steady-state free-precession (SSFP) cine sequence in short- and long-axis views with 25 cardiac phases. Native and post-contrast T1 mapping was performed with a motion-corrected modified look-locker inversion (MOLLI) recovery sequence using the 5 s (3 s) 3 s scheme. T2 mapping was performed with a free-breathing navigator-gated black-blood prepared gradient and spin-echo (GraSE) hybrid sequence in the basal, mid, and apical slices corresponding to the MOLLI sequence. LGE imaging with a phase-sensitive inversion recovery (PSIR) sequence was acquired 10 min after injecting 0.15 mmol/kg of gadoterate meglumine (Dotarem™).

### CMR data analysis

CMR images were post-processed independently and blindly in random order by two investigators (E.T. and H.C.) using CVi42 software (Circle Cardiovascular Imaging Inc). The corresponding short-axis maps were used to carefully delineate endo- and epicardial contours with 10% endo- and epicardial offsets to avoid contamination. Evaluation of LV and RV volumes and LV mass was performed in standard fashion using short-axis cine images [22]. The presence of LGE was visually analyzed. LGE areas were excluded for T1 and ECV quantification. Extracellular volume (ECV) fraction was calculated using a previously validated equation [23]. CMR parameters are presented as the mean of two observers and indexed to the body surface area (BSA).

Myocardial strain values were generated based on cine CMR images using feature-tracking software Segment (version 2.1.R.6108, Medviso) [24]. GLS and global radial strain (GRS) were derived from three long-axis (2-, 3-, and 4-chamber views) cine series, whereas GCS was measured on three short-axis (apical, mid, and basal slices) cine series. Endo- and epicardial contours were manually delineated on end-diastolic images and were then automatically propagated by the software throughout the cardiac cycle, generating myocardial strain [24].

### Statistical analysis

Statistical analysis was performed using SPSS for Windows, version 28.0.1.1 (IBM SPSS Statistics). Graphs were generated using ggplot2 via RStudio (version 2022.12.0.353). Demographic data are presented as median (range), continuous parameters as mean ± SD, and categorical data as absolute numbers and percentages. Normality of continuous data was assessed using the Shapiro–Wilk test. The Man-Whitney-U-Test was used for statistical analysis of demographic data, t-test for metric data, and Fisher’s exact test for categorical variables. Pearson’s correlation or Spearman’s correlation were applied as appropriate. Multivariate regression analyses were executed to analyze the effects of multiple variables on cardiac parameters with LVEF, LV GCS, and RV GCS, respectively, as the dependent variable. All *p* values are two-sided and considered significant at *p* < 0.05.

## Results

### Baseline demographics and tumor characteristics

Median age of survivors was 44 (interquartile range [IQR] 37–52) years. There were no significant differences in age, weight, height, body mass index (BMI), and BSA between survivors and controls (Table 1). The median follow-up time after completion of platinum-based chemotherapy was 10 (IQR 5–15) years (Table 2). Thirty-six percent of survivors had seminoma, and 64% had non-seminoma. The initial disease stage was I in 25%, II in 45%, and III in 30% of survivors, respectively (Table 2). In total, 33 (75%) survivors had received one, and 11 (25%) had received two or more lines of therapy as detailed in Table 2. A total of 10 survivors had received high-dose chemotherapy followed by autologous stem cell transplantation.

**Table 1:** Baseline characteristics of GCC survivors compared to controls

Demographics, median (IQR)	GCC survivors (*n* = 44)	Controls (*n* = 21)	*p* value
Age, years	44 (37–52)	44 (30–52)	0.614
Weight, kg	88 (78–100)	85 (79–94)	0.370
Height, m	1.83 (1.78–1.88)	1.83 (1.75–1.86)	0.457
Body surface area, m^2^	2.1 (2.01–2.25)	2.06 (1.98–2.17)	0.313
Body mass index, kg/m^2^	27 (24–29)	25 (24–29)	0.421

Abbreviations: *GCC*, germ cell cancer; *IQR* interquartile range. *p* values were derived from Man-Whitney-U test

**Table 2:** Tumor characteristics and chemotherapy regimens

	GCC survivors (*n* = 44)
Diagnosis, No. (%)	
Seminoma	16 (36)
Non-Seminoma	28 (64)
Initial disease stage, No. (%)	
I A-B	11 (25)
II A-C	20 (45)
III A-C	13 (30)
Time of follow-up, years, median (IQR)	10 (5–15)
Number of therapy lines, No. (%)	
1	33 (75)
2	6 (14)
3	4 (9)
4	1 (2)
Number of platinum-based chemotherapy cycles, No. (%)	
1	7 (16)
2	4 (9)
3	12 (27)
4	12 (27)
≥ 5	9 (21)
Chemotherapy regimens, No. (%)	
1^st^ line carboplatin AUC7	7 (16)
+ PEB	1 (2)
+ PEI + HD PEI + GOP	1 (2)
1^st^ line PEB^a^	28 (64)
+ HD CE	2 (5)
+ HD CE + TI	2 (5)
+ PEI	2 (5)
+ PEI + HD CE	1 (2)
1^st^ line PEI^b^	5 (11)
+ HD PEI	1 (2)
+ TI + HD CE	1 (2)
1^st^ line cisplatin/etoposide	2 (5)
1^st^ line HD PEI	2 (5)
Total high dose chemotherapy, No. (%)	10 (23)

Abbreviations: *AUC*, area under the curve; *CE*, carboplatin/etoposide; *GCC*, germ cell cancer; *GOP*, gemcitabine/oxaliplatin/paclitaxel; *HD*, high dose; *IQR*, interquartile range; *PEB*, cisplatin/etoposide/bleomycin; *PEI*, cisplatin/etoposide/ifosfamide; *TI*, paclitaxel/ifosfamide

^a^One patient was switched to cisplatin/etoposide, and one was switched to PEI due to bleomycin toxicity

^b^One patient received the first cycle without ifosfamide due to intensive care stay

### Cardiac function, volumes, and mass

**Fig. 2:**
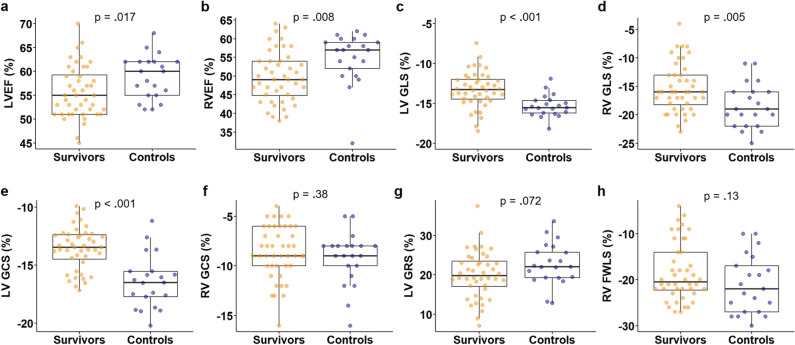
Systolic function in germ cell cancer survivors compared to age-matched controls. Cardiac magnetic resonance imaging was performed in asymptomatic germ cell cancer survivors after platinum-based chemotherapy (*n* = 44) and age-matched healthy controls (*n* = 21). For assessment of systolic function biventricular ejection fraction and myocardial strain was assessed: (**a**) left ventricular ejection fraction (LVEF), (**b**) right ventricular ejection fraction (RVEF), (**c**) LV global longitudinal strain (GLS), (**d**) RV GLS, (**e**) LV global circular strain (GCS), (**f**) RV GCS, (**g**) LV global radial strain (LV GRS), and (**h**) RV free wall longitudinal strain (FWLS). Data are presented as mean ± SD. T-test was performed

Detailed results of functional and volumetric CMR-derived parameters in survivors compared to controls are presented in Supplementary Table 1. LVEF and RVEF were significantly lower in GCC survivors compared to controls (LVEF 56 ± 5% *vs.* 59 ± 5%, *p* = 0.017; RVEF 50 ± 7% *vs.* 55 ± 7%, *p* = 0.008; Fig. 2a and b). Three survivors (7%) showed reduced LVEF below 50%, and 18 (41%) survivors presented with borderline LVEF between 50 and 54%. RV end-systolic volume index (RVESVi), a marker of ventricle remodeling, was significantly higher (45 ± 10 mL/m^2^ *vs.* 38 ± 11 mL/m^2^, *p* = 0.01) and there was a trend towards higher LVESVi in survivors compared to controls (39 ± 9 mL/m^2^ *vs.* 35 ± 10 mL/m^2^, *p* = 0.084; Supplementary Table 1). No significant differences were found for heart rate, LV cardiac index (cardiac output per minute related to BSA), LV mass index, atrial volumes, and ventricular systolic and ventricular end-diastolic volumes, respectively (Supplementary Table 1).

### Myocardial strain

Strain analysis revealed significantly lower myocardial deformation of the left and right ventricle in survivors compared to controls as measured by GLS (LV GLS -13 ± 2% *vs.* -15 ± 1%, *p* < 0.001; RV GLS -15 ± 4% *vs.* -19 ± 4%, *p* = 0.005; Fig. 2c and d) and LV GCS (-14 ± 2% *vs.* -16 ± 2%, *p* < 0.001; Fig. 2e). No significant differences were found for RV GCS, LV radial strain, and RV free wall longitudinal strain (Fig. 2f–h).

### Myocardial tissue characterization

**Fig. 3:**
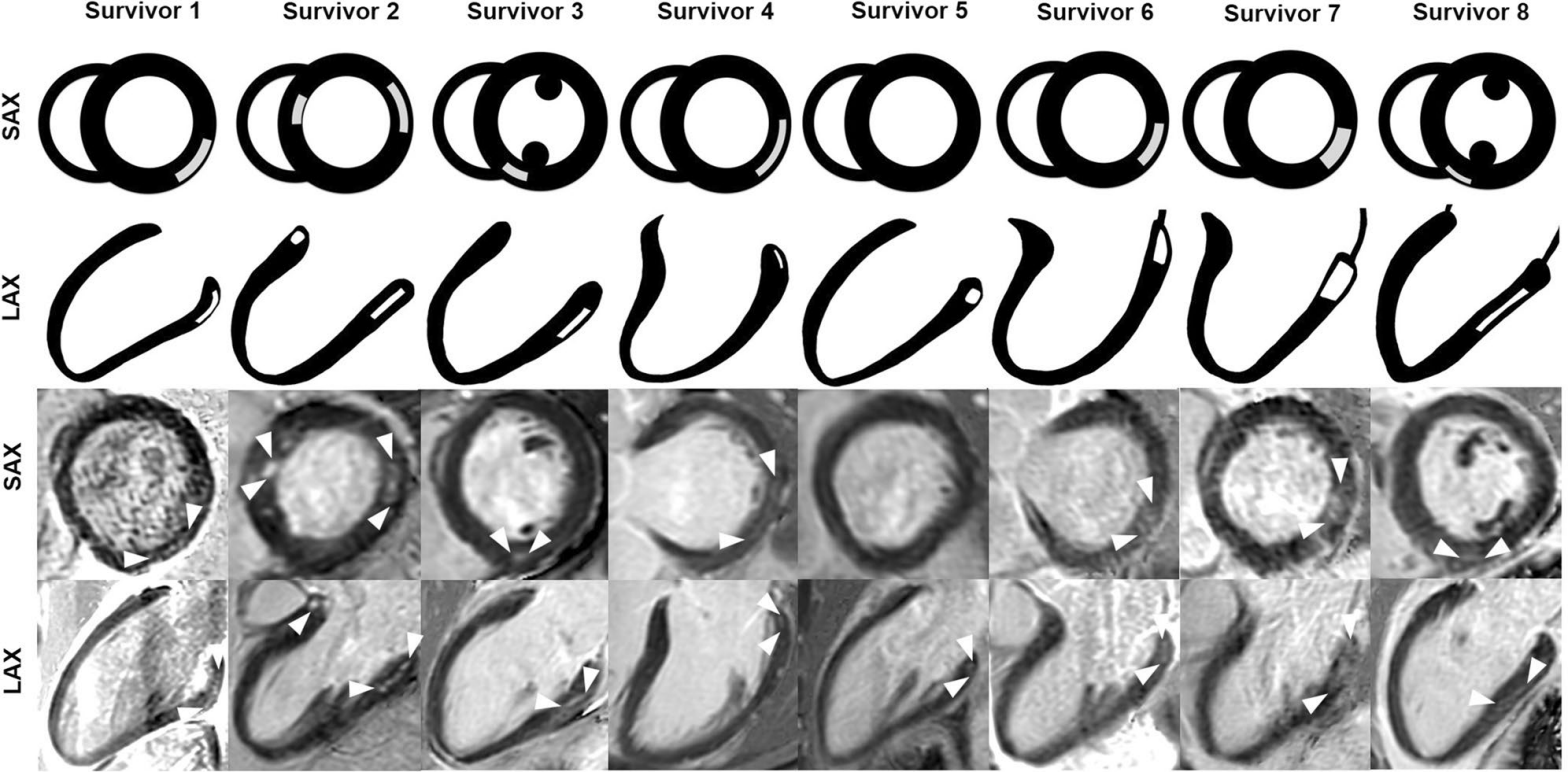
Non-ischemic LGE in germ cell cancer survivors. Myocardial tissue was characterized by cardiac magnetic resonance imaging in asymptomatic germ cell cancer survivors after platinum-based chemotherapy (*n* = 44) and age-matched healthy controls (*n* = 21) by late gadolinium enhancement (LGE) imaging. Short-axis and long-axis images of eight germ cell cancer survivors with non-ischemic LGE are shown. Arrows indicate non-ischemic LGE lesions. LGE lesions are predominantly located at subepicardial and mid-wall left ventricular segments. Two patients had LGE lesions located at the posterior right ventricular insertion point (survivors 3 and 8)

Eight survivors (18%) showed non-ischemic LGE, indicating focal myocardial fibrosis (scar), and one survivor (2%) showed ischemic LGE (Fig. 3 and Supplementary Table 1). Four survivors with non-ischemic LGE had normal LVEF (≥ 55%), and 4 survivors with non-ischemic LGE had borderline LVEF (50–54%) (Table 3).

**Table 3:** Myocardial tissue characteristics in GCC survivors depending on LVEF

	LVEF < 55% (*n* = 21)	LVEF ≥ 55% (*n* = 23)	*p* value
Native T1 time, ms, mean ± SD	1191 ± 22	1211 ± 25	**0.007**
ECV, %, mean ± SD	24.2 ± 1.4	25.6 ± 1.9	**0.008**
LGE presence (non-ischemic), No. (%)	4 (19)	4 (17)	-

Abbreviations: *ECV*, extracellular volume; *LGE*, late gadolinium enhancement; *LVEF*, left ventricular ejection fraction. *p* values were derived from T-test

**Fig. 4:**
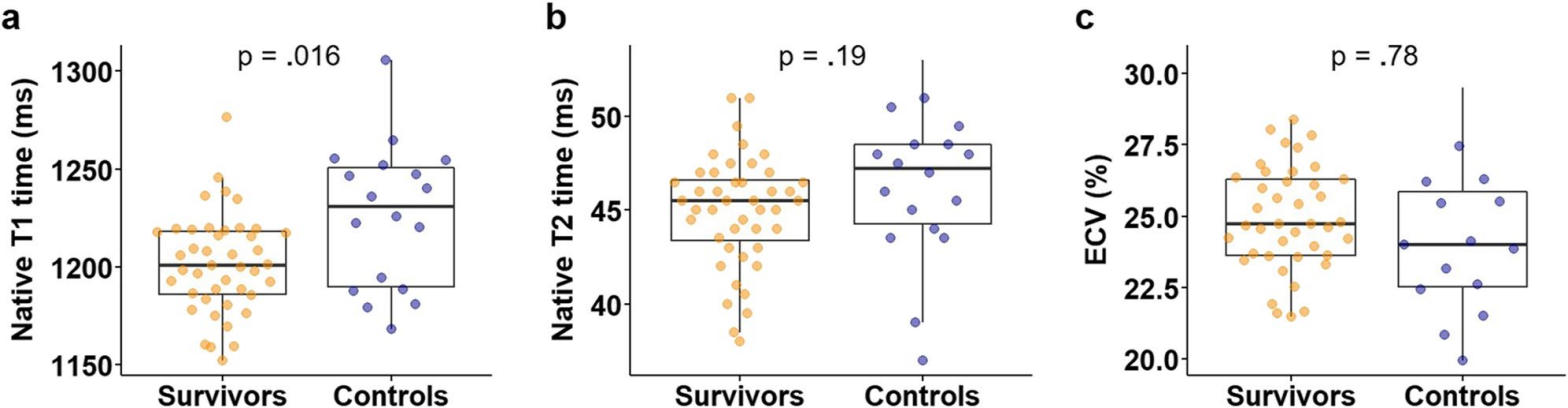
Myocardial tissue alterations in germ cell cancer survivors. Myocardial tissue was characterized by cardiac magnetic resonance imaging in asymptomatic germ cell cancer survivors after platinum-based chemotherapy (*n* = 44) and age-matched healthy controls (*n* = 18) by T1/T2 mapping. **a** Native T1 time, (**b**) native T2 time, and (**c**) extra cellular volume (ECV) were assessed. Data are presented as mean ± SD. T-Test was performed

**Fig. 5:**
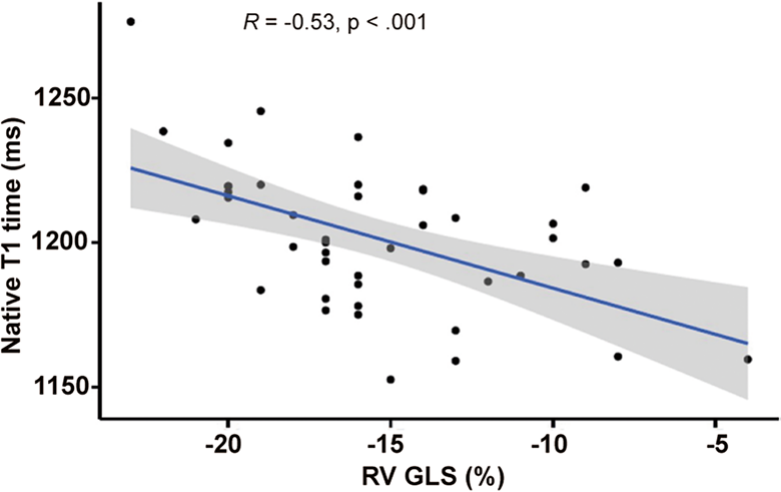
Correlation of native myocardial T1 relaxation time and RV GLS. Myocardial tissue was characterized by cardiac magnetic resonance imaging in asymptomatic germ cell cancer survivors after platinum-based chemotherapy (*n* = 44) by T1/T2 mapping. Pearson’s correlation was used to analyze the association of native myocardial T1 relaxation time and right ventricular global longitudinal strain (RV GLS)

Myocardial native T1 mapping revealed lower T1 relaxation times in survivors compared to controls (1202 ± 25 ms *vs.* 1226 ± 37 ms, *p* = 0.016; Fig. 4a). Survivors with decreased or borderline LVEF below 55% had lower myocardial T1 values when compared to those with LVEF ≥ 55% (1191 ± 22 ms *vs.* 1211 ± 25 ms, *p* = 0.007; Table 3). A moderate positive correlation was found between native T1 values and LVEF (*r*(41) = 0.346, *p* = 0.023), and RVEF (*r*(41) = 0.314, *p* = 0.04), respectively. There was a moderate negative correlation between native T1 values and LV GLS (*r*(41) =  −0.341, *p* = 0.025), and a strong negative correlation between native T1 values and RV GLS (*r*(41) =  −0.534, *p* < 0.001, Fig. 5), respectively. T2 values and ECV did not differ between groups (Fig. 4b and c). However, survivors with LVEF below 55% had significantly lower percentage of ECV compared with survivors with normal LVEF (24.2 ± 1.4% *vs.* 25.6 ± 1.9%, *p* = 0.008; Table 3).

### Cardiac parameters in association with chemotherapy dose

**Fig. 6:**
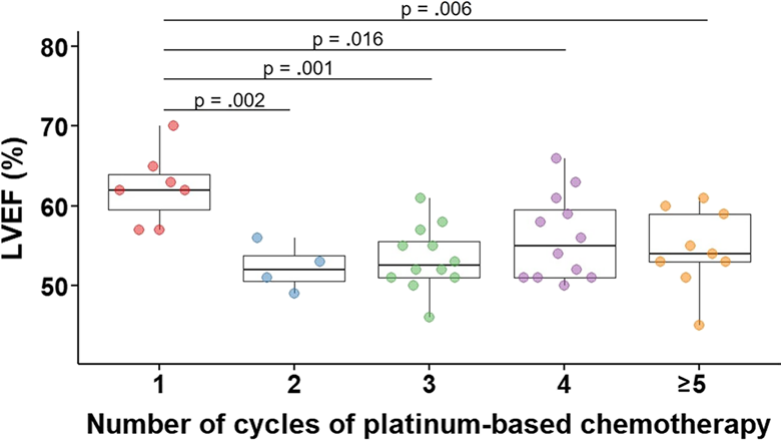
LVEF impairment after two cycles of platinum-based chemotherapy. Graph shows left ventricular ejection fraction (LVEF) in germ cell cancer survivors (*n* = 44) measured by cardiac magnetic resonance imaging for subgroups depending on the number of platinum-based chemotherapy cycles received. T-test was applied

Correlation of cumulative platinum dose and LVEF showed a drop in LVEF after merely two cycles of platinum-based combination chemotherapy (Fig. 6). Therefore, cardiac parameters and clinical characteristics were compared between survivors who received one cycle of chemotherapy (carboplatin AUC7 or cisplatin 100 mg/m^2^, n = 7) and those who received at least two cycles of platinum-based combination chemotherapy (cisplatin ≥ 200 mg/m^2^ ± carboplatin, *n* = 37).

Survivors who had received ≥ 2 cycles of chemotherapy showed significantly higher body weight and BSA (Table 4). No differences were found for anamnestic cardiovascular risk factors or medication. The laboratory analysis revealed significantly impaired renal functional parameters and lower testosterone levels, and a trend towards higher cholesterol levels in survivors after ≥ 2 cycles of platinum-based chemotherapy. No significant differences were found for cardiac biomarkers Troponin I and NT-proBNP or ferritin values (Table 4). Two survivors who had received > 2 cycles of platinum-based combination chemotherapy showed elevated high-sensitive Troponin I. Both individuals presented with non-ischemic LGE not fulfilling criteria for acute myocarditis; acute myocardial infarction was ruled out in both individuals.

**Table 4:** Clinical characteristics and cardiac parameters depending on number of platinum-based chemotherapy cycles

	Platinum-based chemotherapy		*p* value
	1 cycle (*n* = 7)	≥ 2 cycles (*n* = 37)	
Demographics, median (IQR)			
Age, years	44 (37–54)	44 (37–52)	0.832
Follow-up, years	10 (6–12)	10 (5–15)	0.919
Height, m	1.78 (1.77–1.86)	1.83 (1.80–1.89)	0.212
Weight, kg	77 (74–85)	90 (82–100)	**0.023**
BMI, kg/m^2^	24 (23–27)	27 (24–29)	0.111
BSA, m^2^	2.02 (1.91–2.03)	2.13 (2.13–2.23)	**0.025**
Medical history, No. (%)			
Dyslipidemia	2 (29)	6 (16)	0.593
Diabetes mellitus	-	2 (5)	
Arterial hypertension	1 (14)	7 (19)	0.624
Former or active smoker	3 (43)	17 (46)	0.606
Antihypertensive Medication	1 (14)	8 (22)	0.557
Lipid-lowering drug	1 (14)	1 (3)	0.296
Cardiac parameters, mean ± SD			
Heart rate, beats/min	66 ± 15	68 ± 13	0.683
Left heart			
LVEF, %	62 ± 5	54 ± 5	** < 0.001**
> 55%, No. (%)	7 (100)	16 (43)	**0.02**
50–54%, No. (%)		18 (49)	-
< 50%, No. (%)		3 (8)	-
LV mass index, g/m^2^	53 ± 5	59 ± 5	0.270
LVEDVi, mL/m^2^	85 ± 13	87 ± 14	0.733
LVESVi, mL/m^2^	32 ± 6	40 ± 8	**0.023**
LVSVi, mL/m^2^	53 ± 9	47 ± 8	0.072
LAEDVi, mL/m^2^	14 ± 4	16 ± 7	0.398
LAESVi, mL/m^2^	30 ± 9	33 ± 9	0.442
Right heart			
RVEF, %	53 ± 7	49 ± 7	0.2
RVEDVi, mL/m^2^	92 ± 16	89 ± 15	0.526
RVESVi, mL/m^2^	43 ± 10	45 ± 10	0.643
RVSVi. mL/m^2^	49 ± 10	44 ± 9	0.138
RAEDVi, mL/m^2^	28 ± 8	25 ± 8	0.379
RAESVi, mL/ m^2^	44 ± 6	41 ± 12	0.501
Strain parameters			
LV GLS, %	-14 ± 2	-13 ± 2	0.339
LV GCS, %	-16 ± 2	-13 ± 2	** < 0.001**
LV GRS, %	20 ± 8	20 ± 6	0.865
RV GLS, %	-16 ± 6	-15 ± 4	0.642
RV FWLS, %	-18 ± 9	-19 ± 6	0.740
RV GCS, %	-10 ± 2	-8 ± 3	**0.046**
LGE presence, No. (%)			
Non-ischemic LGE	-	8 (22)	
Ischemic LGE	-	1 (2)	
Mapping parameters			
Native global T1, ms	1207 ± 21	1201 ± 26	0.531
Native global T2, ms	45 ± 3	45 ± 3	0.707
ECV, %	26 ± 2	25 ± 2	0.053
Laboratory Findings, mean ± SD			
Cholesterol (mg/dl)	181 ± 31	203 38	0.082
HDL (mg/dl)	50 ± 12	46 ± 12	0.207
LDL (mg/dl)	104 ± 28	123 ± 33	0.081
Testosterone (µg/L)	6 ± 3	4 ± 2	**0.002**
Troponin (pg/mL)	3 ± 2	9 ± 25	0.535
NT-proBNP (ng/l)	45 ± 12	49 ± 33	0.727
Creatinine (mg/dl)	0.9 ± 0.1	1.1 ± 0.2	**0.027**
GFR (mL/Min)	100 ± 16	87 ± 16	**0.025**
Ferritin (µg/l)	120 ± 62	151 ± 68	0.316

Abbreviations: *BMI*, body mass index; *BSA*, body surface area; *ECV*, extracellular volume; *FWLS*, free wall longitudinal strain; *GCC*, germ cell cancer; *GCS*, global circumferential strain; *GFR*, glomerular filtration rate; *GLS*, global longitudinal strain; *GRS*, global radial strain; *HDL*, high-density lipoprotein; *IQR*, interquartile range; *LA*, left atrial; *LAEDVi*, left atrial end-diastolic volume index; *LAESVi*, left atrial end-systolic volume index; *LDL* low-density lipoprotein; *LGE*, late gadolinium enhancement; *LV*, left ventricular; *LVEDVi*, left ventricular end-diastolic volume index; *LVEF*, left ventricular ejection fraction; *LVESVi*, left ventricular end-systolic volume index; *LVSVi*, left ventricular stroke volume index; *RA*, right atrial; *RAEDVi*, right atrial end-diastolic volume index; *RAESVi*, right atrial end-systolic volume index; *RV*, right ventricular; *RVEDVi*, right ventricular end-diastolic volume index; *RVESVi*, right ventricular end-systolic volume index

*p* values were derived from T-test for continuous data, from Man-Whitney-U test for demographic data and Fisher’s exact test for categorical variables, respectively. Bold values indicate statistical significance at the* p *< 0.05 level

LVEF was significantly lower in survivors after ≥ 2 cycles of platinum-based combination chemotherapy compared to survivors who had received one cycle of chemotherapy (54 ± 5% *vs.* 62 ± 5%, *p* < 0.001; Table 4). Strain analysis showed a significant decrease in LV and RV GCS after ≥ 2 cycles of chemotherapy (LV GCS -13 ± 2% *vs.* -16 ± 2%, *p* < 0.001; RV GCS -8 ± 3% *vs.* -10 ± 2%, *p* = 0.046; Table 4), indicating decreased myocardial deformation after higher doses of chemotherapy. No significant changes were found for RVEF, LV GLS, and RV GLS, native T1 and T2 times, respectively. Notably, all survivors with focal LGE had received ≥ 2 cycles of platinum-based combination chemotherapy (Table 4). The multivariate regression analysis showed that the association of chemotherapy cycles with LVEF, LV GCS, and RV GCS, respectively, was independent of testosterone levels, renal function, body weight, and BSA.

Cardiac parameters did not differ between survivors who had received high-dose chemotherapy and autologous stem cell transplantation and those without high-dose chemotherapy.

### Cardiac parameters in association with clinical and laboratory parameters

GCC survivors with anamnestic arterial hypertension had lower native T1 values (1179 ± 19 ms *vs.* 1207 ± 24 ms, *p* = 0.003). Anamnestic dyslipidemia or smoking status, age, and time of follow-up were not associated with differences in cardiac parameters. A weak negative correlation was found for LV GLS and HDL levels (*r*(42) =  −0.347, *p* = 0.021). Beyond that, systolic functional parameters and T1 values did not correlate with levels of cholesterol, LDL, HDL, and testosterone, respectively.

## Discussion

To the best of our knowledge, this is the first study to extensively characterize cardiac alterations in long-term survivors of GCC using CMR imaging. The study has three major findings: (1) We found attenuated systolic function in GCC survivors almost 10 years after therapy completion as measured by EF and strain analysis after only two cycles of platinum-based combination chemotherapy; (2) tissue characterization revealed that focal myocardial fibrosis detected by LGE was common in GCC survivors; and (3) survivors presented with mildly decreased native T1 values compared to controls.

Previous studies investigating the cardiac function of GCC survivors mainly used echocardiography. A recent study by Bjerring et al performed echocardiography including speckle-tracking derived strain analysis 30 years after cisplatin-based chemotherapy for GCC and found no differences in biventricular systolic function between survivors and controls [9]. The current study using CMR, including strain analysis based on a feature-tracking technique, demonstrated subclinical attenuation of systolic function after cisplatin-based chemotherapy after a median follow-up time of 10 years but not after one course of carboplatin. This functional impairment was observed after a threshold of two cycles of cisplatin-based combination chemotherapy (at ≥ 200 mg/m^2^ cumulative dose) without further dose-dependent decline beyond that, although data on this association are clearly still limited. These findings indicate that platinum-based chemotherapy has more extensive effects on the heart than known so far. This observation is supported by a previous in vivo study that described cisplatin-induced development of LV dysfunction and depressed cardiomyocyte contraction in mice that was associated with mitochondrial abnormalities, endoplasmic reticulum stress response, and apoptosis [25]. However, considering the results of Bjerring et al with a longer follow-up of 30 years, the observed subclinical changes in systolic function might not necessarily progress to clinical manifestation of heart failure [9]. In a population-based cohort study, mortality due to non-ischemic cardiac disease was increased in GCC survivors following combined chemo- and radiotherapy but not after chemotherapy alone [26]. Nevertheless, in the current study cohort 4 out of 45 asymptomatic survivors had decreased LVEF. Identifying patients with clinically asymptomatic structural and functional cardiac abnormalities provides an opportunity to initiate lifestyle modifications and pharmacological therapy that may prevent or delay progression to symptomatic heart failure [27]. Therefore, this study raises the question whether specific cardiological examinations should be included into the follow-up routine of GCC survivors after ≥ 200 mg/m^2^ cumulative cisplatin.

The current study revealed a high prevalence (18%) of focal myocardial fibrosis as identified by mid-wall/subepicardial LGE. Previous studies reported non-ischemic LGE in 4–9% of cancer patients and in 2.8–4% of healthy individuals [28–30]. The clinical significance of non-ischemic LGE in cancer survivors remains unclear. In general, the presence of LGE has a prognostic value in patients with preexisting heart disease, whereas subjects with minor non-ischemic LGE and no further structural or functional cardiac abnormalities are considered to have a favorable outcome [20, 31].

The lower T1 values indicated myocardial tissue changes after platinum-based chemotherapy compared to controls. A decrease of native T1 values in patients shortly after anthracycline administration has previously been described and identified as a predictor of subsequent anthracycline-induced cardiomyopathy [32]. A decrease in native T1 is described in iron or lipid overload [33]. Interestingly, we found that worse systolic functional parameters in survivors correlated with a decrease in native T1 values, pointing toward functional consequences of these tissue alterations. It remains unclear which myocardial tissue alterations underlie the detected native T1 decrease in GCC survivors. Ferritin values were within the normal range in this study cohort, excluding iron overload. Also, decreased T1 values, no differences in ECV compared to healthy controls, and lower ECV in survivors with LVEF below 55% argue against diffuse interstitial fibrosis in the myocardium of GCC survivors. Whether the reported decrease in T1 values is due to ultrastructural mitochondria abnormalities and accumulation of membranous debris within and dilation of the endoplasmatic reticulum in cardiomyocytes as described in cisplatin-treated mice remains to be elucidated.

Lower T1 values in patients with anamnestic arterial hypertension and association of lower HDL with worse LV GLS indicate that the observed functional changes and tissue alterations might be a secondary sequela. However, these associations were not robustly detected for all functional parameters. Based on this study, we cannot draw a conclusion on whether the observed cardiac effects are a result of direct chemotherapy-induced cardiotoxicity or rather a secondary result of metabolic changes or arterial hypertension.

Our study has some shortcomings. CMR is the gold standard for the assessment of systolic function and tissue characterization, a major limitation is that diastolic function cannot be validly assessed. Furthermore, this study does not allow to differentiate which chemotherapy agent is the cause of the observed cardiac effects. Even though almost every chemotherapy cycle contained a platinum agent, most survivors had also received several cycles of bleomycin and etoposide. However, survivors treated with only one cycle of carboplatin single agent did not seem to be at increased risk. The longitudinal studies with baseline CMR examinations and a longer follow-up are needed in order to elucidate the clinical significance and prognostic value of the subclinical systolic impairment observed in this cohort. Moreover, further studies are warranted to validate the finding of decreased T1 time in this survivorship group, as observed changes were rather small.

In conclusion, this study revealed for the first time that subclinical attenuation of LV and RV systolic function characterized by decreased biventricular ejection fraction and myocardial deformation, and changes in myocardial tissue characteristics can be detected in asymptomatic long-term GCC survivors following platinum-based chemotherapy. This study emphasizes the need for more in-depth investigations into the risk of cardiotoxicity associated with PEB (cisplatin/etoposide/bleomycin) chemotherapy and points to the importance of long-term cardiovascular follow-up in GCC survivors as well as education programs to avoid additional cardiac risk behavior in this survivorship population.

### Supplementary Information

Below is the link to the electronic supplementary material.

Supplementary file1 (PDF 49 KB)

## Abbreviations

AUC: Area under the curve
BSA: Body surface area
CMR: Cardiac magnetic resonance
ECV: Extracellular volume
EF: Ejection fraction
GCC: Germ cell cancer
GCS: Global circumferential strain
GLS: Global longitudinal strain
GRS: Global radial strain
HDL: High-density lipoprotein
IQR: Interquartile range
LDL: Low-density lipoprotein
LGE: Late gadolinium enhancement
LV: Left ventricular
RV: Right ventricular

## Declarations

### Guarantor

The scientific guarantor of this publication is Antonia Beitzen-Heineke.

### Statistics and biometry

Dr. Linda Krause (University Medical Center Hamburg-Eppendorf, Center for Experimental Medicine, Institute of Medical Biometry and Epidemiology) kindly provided statistical advice for this study.

### Methodology

• prospective

• case–control study

• performed at one institution
