# Supplementary material for: Long-term cardiotoxicity in germ cell cancer survivors after platinum-based chemotherapy: cardiac MR shows impaired systolic function and tissue alterations
Source: Eur Radiol. 2023 Nov 20;34(6):4102–12. doi: 10.1007/s00330-023-10420-w (PMC11166766; doi:10.1007/s00330-023-10420-w)
Supplement: Supplementary file 1 — Supplementary file1 (PDF 49 KB) [file 330_2023_10420_MOESM1_ESM.pdf]

Long-term cardiotoxicity in germ cell cancer survivors after platinum-based chemotherapy: Cardiac MR shows impaired systolic function and tissue alterations

Electronic Supplementary Material

Supplementary Table 1. Cardiac parameters of GCC survivors compared to controls.

	**GCC survivors** (n= 44)	**Controls** (n= 21)	***p* value**
Left heart, mean ±SD			
Heart rate, beats/min	68 ±13	67 ±13	0.379
LV cardiac index, L/min/m²	3.2 ±0.69	3.2 ±0.71	0.443
LVEF, %<br>>55%, No. (%)<br>50%-54%, No. (%)<br><50%, No. (%)	56 ±5<br>23 (52)<br>18 (41)<br>3 (7)	59 ±5<br>18 (86)<br>3 (14)<br>-	0.017<br>-<br>-<br>-
LV mass index, g/m²,	58 ±11	58 ±12	0.992
LVEDVi, mL/m²	87 ±14	83 ±18	0.388
LVESVi, mL/m²	39 ±9	35 ±10	0.084
LVSVi, mL/m²	48 ±8	49 ±9	0.793
LAEDVi, mL/m²	16 ±7	13 ±4	0.133
LAESVi, mL/m²	33 ±9	30 ±8	0.2
Right heart, mean ±SD			
RVEF, %	50 ±7	55 ±7	0.008
RVEDVi, mL/m²	89 ±15	83 ±15	0.111
RVESVi, mL/m²	45 ±10	38 ±11	0.01
RVSVi, mL/m²	44 ±10	45 ±9	0.789
RAEDVi, mL/m²	25 ±8	23 ±8	0.332
RAESVi, mL/ m²	42 ±11	39 ±15	0.446
Strain parameters, mean ±SD			
LV GLS, %	-13 ±2	-15 ±1	<0.001
LV GCS, %	-14 ±2	-16 ±2	<0.001
LV GRS, %	20 ±6	23 ±5	0.072
RV GLS, %	-15 ±4	-19 ±4	0.005
RV FWLS, %	-19 ±6	-21 ±6	0.13
RV GCS, %	-9 ±3	-9 ±3	0.38
LGE presence, No. (%)			
Non-ischemic LGE	8 (18)	-	-
Ischemic LGE	1 (2)	-	-
Mapping parameters, mean ±SD	(n=44)	(n=18)	
Native global T1, ms	1202 ±25	1226 ±37	0.016
Native global T2, ms	45 ±3	46 ±4	0.19
ECV, %	25 ±2	24 ±3	0.78

Abbreviations: ECV, extracellular volume; FWLS, free wall longitudinal strain; GCC, germ cell cancer; GCS, global circumferential strain; GLS, global longitudinal strain; GRS, global radial strain; LA, left atrial; LAEDVi, left atrial end-diastolic volume index; LAESVi, left atrial end-systolic volume index; LGE, late gadolinium enhancement; LV, left ventricular; LVEDVi, left ventricular end-diastolic volume index; LVEF, left ventricular ejection fraction; LVESVi, left ventricular end-systolic volume index; LVSVi, left ventricular stroke volume index; RA, right atrial; RAEDVi, right atrial end-diastolic volume index; RAESVi, right atrial end-systolic volume index; RV, right ventricular; RVEDVi, right ventricular end-diastolic volume index; RVESVi, right ventricular end-systolic volume index.
